# Mechanical Cues: Bidirectional Reciprocity in the Extracellular Matrix Drives Mechano-Signalling in Articular Cartilage

**DOI:** 10.3390/ijms222413595

**Published:** 2021-12-18

**Authors:** Sophie Jane Gilbert, Cleo Selina Bonnet, Emma Jane Blain

**Affiliations:** Biomechanics and Bioengineering Centre Versus Arthritis, School of Biosciences, Cardiff University, Cardiff CF10 3AX, UK; gilbertsj1@cardiff.ac.uk (S.J.G.); bonnetcs@cardiff.ac.uk (C.S.B.)

**Keywords:** articular cartilage, mechanobiology, extracellular/pericellular matrix, homeostatic balance, osteoarthritis

## Abstract

The composition and organisation of the extracellular matrix (ECM), particularly the pericellular matrix (PCM), in articular cartilage is critical to its biomechanical functionality; the presence of proteoglycans such as aggrecan, entrapped within a type II collagen fibrillar network, confers mechanical resilience underweight-bearing. Furthermore, components of the PCM including type VI collagen, perlecan, small leucine-rich proteoglycans—decorin and biglycan—and fibronectin facilitate the transduction of both biomechanical and biochemical signals to the residing chondrocytes, thereby regulating the process of mechanotransduction in cartilage. In this review, we summarise the literature reporting on the bidirectional reciprocity of the ECM in chondrocyte mechano-signalling and articular cartilage homeostasis. Specifically, we discuss studies that have characterised the response of articular cartilage to mechanical perturbations in the local tissue environment and how the magnitude or type of loading applied elicits cellular behaviours to effect change. In vivo, including transgenic approaches, and in vitro studies have illustrated how physiological loading maintains a homeostatic balance of anabolic and catabolic activities, involving the direct engagement of many PCM molecules in orchestrating this slow but consistent turnover of the cartilage matrix. Furthermore, we document studies characterising how abnormal, non-physiological loading including excessive loading or joint trauma negatively impacts matrix molecule biosynthesis and/or organisation, affecting PCM mechanical properties and reducing the tissue’s ability to withstand load. We present compelling evidence showing that reciprocal engagement of the cells with this altered ECM environment can thus impact tissue homeostasis and, if sustained, can result in cartilage degradation and onset of osteoarthritis pathology. Enhanced dysregulation of PCM/ECM turnover is partially driven by mechanically mediated proteolytic degradation of cartilage ECM components. This generates bioactive breakdown fragments such as fibronectin, biglycan and lumican fragments, which can subsequently activate or inhibit additional signalling pathways including those involved in inflammation. Finally, we discuss how bidirectionality within the ECM is critically important in enabling the chondrocytes to synthesise and release PCM/ECM molecules, growth factors, pro-inflammatory cytokines and proteolytic enzymes, under a specified load, to influence PCM/ECM composition and mechanical properties in cartilage health and disease.

## 1. Introduction

**Figure 1:**
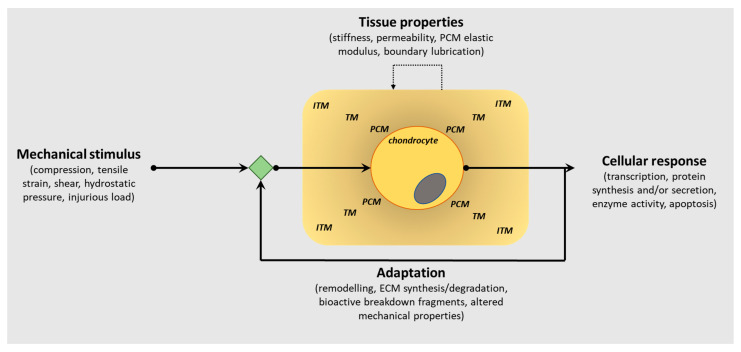
Schematic representation of the bidirectional reciprocity of the pericellular/extracellular matrix (ECM) in chondrocyte mechano-signalling and articular cartilage homeostasis. This bidirectionality within the extracellular matrix (ECM) is critically important in enabling the chondrocytes to sense load application, including altered loading patterns, but also to influence PCM/ECM composition as a consequence of mechanical cues to regulate the structural, biomechanical and functional characteristics of articular cartilage in tissue health and pathology. (PCM, pericellular matrix; TM, territorial matrix; ITM, interterritorial matrix).

Articular cartilage, located at the ends of diarthrodial joints, has a unique biochemical composition that confers its functional properties, the principal function being the dissipation of mechanical forces evenly across the joint surface. The composition of the extracellular matrix (ECM) in articular cartilage is critical to its function, and it is well known that the presence of proteoglycans embedded within a collagen fibrillar network provides biomechanical resilience. Furthermore, the spatial distribution of these ECM molecules within the tissue also facilitates its capability to withstand mechanical loads, as well as perceive biomechanical signals transducing them to the chondrocytes (only cell type in cartilage) to effect cellular responses. This bidirectionality within the ECM is critically important in enabling the chondrocytes to sense load application, including altered loading patterns, but also to influence ECM composition as a consequence of mechanical cues (Figure 1).

**Figure 2:**
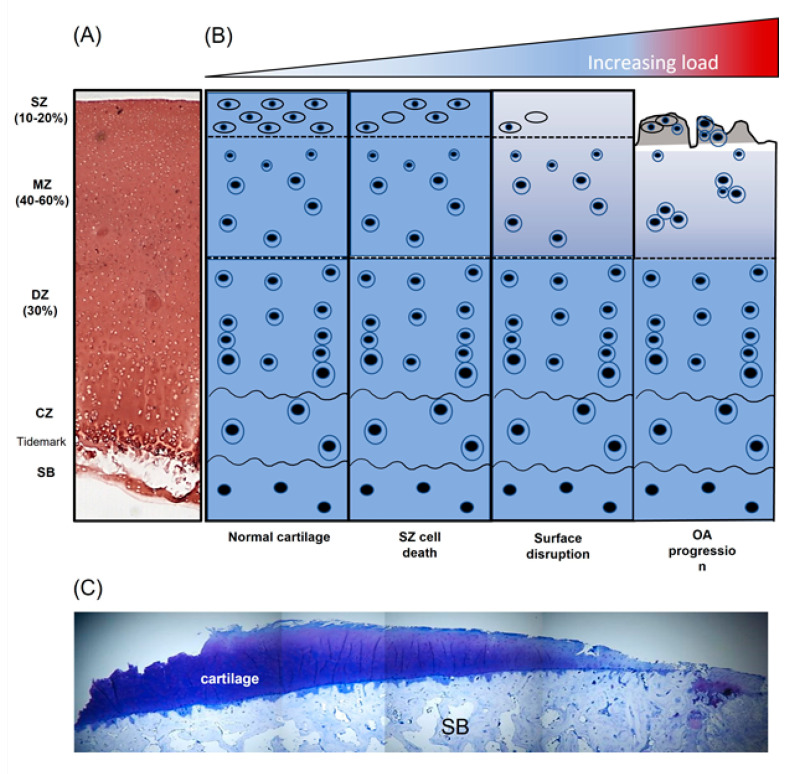
Diagrammatic overview demonstrating the influence of non-physiological load on articular cartilage composition and structure. (**A**) Safranin-O stained section of immature bovine articular cartilage, (**B**) schematic representation of articular chondrocyte morphology and cartilage disruption with increasing pathological load and (**C**) toluidine-blue -stained section of knee articular cartilage from a patient with end-stage osteoarthritis (OA) (adapted from [14]). SZ, superficial zone; MZ, middle zone; DZ, deep zone; CZ, calcified zone; SB, subchondral bone.

Articular cartilage is unique in being avascular, aneural and alymphatic and is largely comprised of a dense ECM interspersed with chondrocytes, which account for only 5–10% of tissue volume [1]. It is a multiple layer structure consisting of a superficial zone (SZ), middle zone, deep zone, tidemark and calcified layer (Figure 2A); the highly organised distribution of the ECM constituents within these layers and their interactions provides a porous and permeable tissue that facilitates the long-term load-bearing capabilities of the joint [2,3,4]. The principal component of the cartilage ECM is water (60–85% by wet weight), whereas the remaining mass comprises a crosslinked network of type II collagen (15–22% by wet weight), proteoglycans (4–7% by wet weight) and smaller amounts of other collagen types, e.g., types VI, IX, X and XI, and non-collagenous proteins, e.g., fibronectin (FN) and tenascin [5,6]. Produced in large amounts by synoviocytes and SZ chondrocytes, lubricin, encoded by the proteoglycan 4 (*Prg4*) gene, colocalises with cartilage oligomeric protein (COMP) and binds FN and type II collagen [7]. It contributes to the highly lubricated cartilage surface [8] and is critical for SZ integrity since mice lacking *Prg4* exhibit a loss of cartilage structure, change in stiffness and frictional properties [9] and develop cartilage wear as they age [10,11]. Mechanistically this may occur via the nuclear factor-κB–matrix metalloproteinase 9–transforming growth factor beta (NFκB–MMP9–TGFβ) axis since lubricin is increased by TGFβ and lubricin suppresses SZ MMP9 expression preventing chondrocyte differentiation [7,12]. Recently, β-catenin signalling has also been linked to lubricin’s role in maintaining cartilage surface integrity since deletion of β-catenin in *Prg4*-expressing SZ cells leads to failure of the SZ and enhanced progression of osteoarthritis (OA) [13].

## 2. Composition and Localisation of ECM Components Are Highly Organised in Articular Cartilage and Confer Biomechanical Functionality

The ECM of articular cartilage is comprised largely of two matrix compartments, the fibrillar collagen network and the extrafibrillar matrix. These are connected by a number of perifibrillar adaptor proteins and collectively provide stability and structural integrity (reviewed in [15]).

### 2.1. Collagens 

The collagen fibrils of articular cartilage are largely composed of type II (>90%), XI (~3%) and IX (~1%) collagens [16,17] and are densely packed and orientated to allow them to resist the forces imposed by articulation [18]. The N-terminal short arm of type IX collagen projects into the perifibrillar space to provide binding sites to other matrix molecules such as FN [4] whilst the long arm is orientated towards the fibril to allow covalent interactions with type II collagen and other type IX molecules [19,20,21,22]. Type III collagen which colocalises with type II collagen [23,24,25] and type IV [5,26,27] is also found in small amounts within the pericellular matrix (PCM) and is thought to play a role in regulating fibril diameter and modifying existing fibril networks [27,28]. Type VI collagen anchors the chondrocytes to the ECM, mediating cell–matrix interactions and transducing mechanical cues—discussed in detail below [29,30,31].

### 2.2. Proteoglycans

The most abundant proteoglycan (PG) by mass in cartilage is aggrecan; it is heavily glycosylated with more than 100 negatively charged sulphated GAGs including chondroitin sulphate and keratan sulphate. Versican is also present as an aggregating chondroitin sulphate PG in developing cartilage but with levels declining with age [32,33]. Both aggrecan [34] and versican [32] form large aggregates, via link proteins, with the glycosaminoglycan (GAG) hyaluronan which is critical for the maintenance of the pericellular environment surrounding the cell [35]. These large aggregates attract solutes generating an osmotic swelling pressure which is critical to counteract compressive forces during movement [36]. Since versican interacts with fibrillin-1 which binds latent TGFβ-binding protein 1 [37], it has been proposed that versican plays a role in TGFβ signalling [32]. In addition to the large aggregating PGs, non-aggregating PGs are present including the small leucine-rich proteoglycans (SLRPs), biglycan (BGN) and decorin (DCN) and fibromodulin. The PCM consists of high concentrations of the hyaluronan–aggrecan link protein complexes and BGN and DCN [38,39] and is associated with rapid rates of aggrecan turnover [40], proteoglycan deposition [41] and the greatest sensitivity to static and dynamic loads [42].

### 2.3. Perifibrillar Adapter Proteins

Perifibrillar adapter proteins, including matrilin-1-4 (reviewed in [43]), DCN [39,44], COMP [6,45] and type IX collagen [22,46], are essential for healthy cartilage, playing important roles in regulating fibril diameter in immature cartilage, stabilising the ECM network in adult cartilage and interconnecting the fibrillar and extrafibrillar matrix [43,44,47,48]. All four matrilins are expressed in adult cartilage at low levels and bind to various ECM components [49]; matrilin-3 forms complexes with matrilin-1 and -4 that are linked via BGN and DCN to type VI collagen microfibrils (reviewed in [43]). Whilst individual matrilin knockouts have mild phenotypes with other matrilins compensating for their loss [50], quadruple knockout mice exhibit a severe osteoarthritic phenotype with age revealing a role for matrilins in protecting from spontaneous OA [49]. The importance of COMP has been highlighted by a number of studies which found that it catalyses fibril formation [51] and, in adult cartilage, stabilises the network [6] by binding to type IX collagen [6,52], matrilin-3 and -4 [45,46] and connecting the collagen network to aggrecan via BGN and DCN [53] (reviewed in [43]). The complex of collagen IX, matrilin-3 and COMP is essential for cartilage matrix stability since mutations in these genes cause multiple epiphyseal dysplasia with patients developing early-onset OA [54]. In addition, loss of type IX collagen results in reduced integration of COMP and matrilin-1, -3 and -4 into the ECM [46,55], a softer cartilage matrix [56] and increased fibril diameter [46,55].

## 3. The Pericellular Matrix and Its Role in Chondrocyte Mechanotransduction

**Figure 3:**
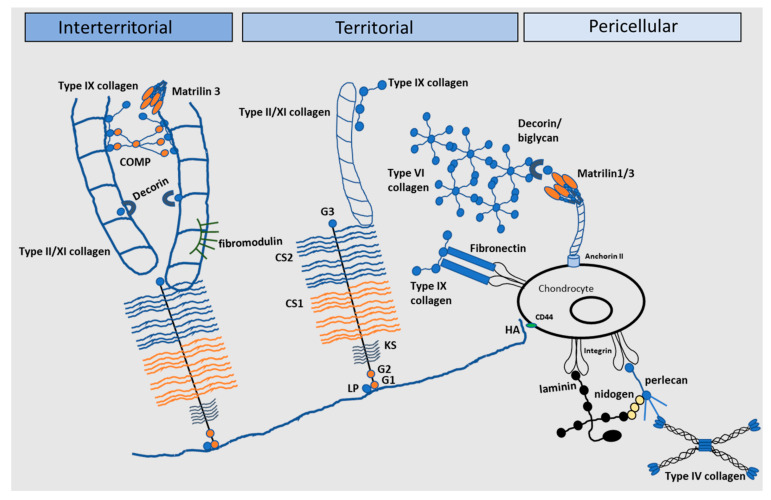
The extracellular matrix of articular cartilage. The extracellular matrix (ECM) of articular cartilage is comprised largely of collagen and proteoglycans, with smaller amounts of other non-collagenous proteins and glycoproteins. Large proteoglycan aggregates such as aggrecan interact with collagens and contain chondroitin sulphate (CS) and keratan sulphate (KS), covalently linked to hyaluronic acid (HA) by link protein (LP). Matrilin-1, -3 and -4, decorin, COMP and type IX collagen link the type II/XI collagen fibrils and mediate interactions between the collagen network and the aggrecan matrix (G globular) (Adapted from [81]).

Organisation of the chondrocytes within the tissue’s ECM is central to its biomechanical functionality [39,57,58,59,60,61]. Articular cartilage ECM is split into two main areas: a PCM and a territorial matrix (TM)/interterritorial matrix (ITM) region (Figure 3). The highly specialised PCM is a 2–4 µm thick region compositionally and structurally distinct from the surrounding ECM; chondrocytes encapsulated within the PCM collectively form the ‘chondron’. Interestingly, chondrons are orientated relative to the direction of local mechanical strains and demonstrate a depth-dependent polarised morphology [39]. Experimental studies and theoretical models have demonstrated that the PCM facilitates the transduction of both biomechanical and biochemical signals to the residing chondrocytes [58,59,60,61], thereby regulating the process of mechanotransduction in the chondrocyte [30,57,62,63,64]. To facilitate this, the PCM is softer than the rest of the ECM [39,65] but has a higher modulus than the cell [66] acting as a biomechanical buffer [67] to control the deformation applied to the cell [68]. In addition, the PCM regulates chondrocyte phenotype [69], with cell survival directly dependant on the interactions between the chondrocyte and the PCM [70,71], and acts as a biological reservoir sequestering growth factors and signalling molecules [72,73,74,75]. The unique structure of the PCM enables it to function in these important roles; it is rich in PGs (e.g., aggrecan, hyaluronan, DCN, perlecan, BGN), collagens (IX, VI collagen), basement membrane proteins (laminin, nidogens, collagen IV) and non-collagenous glycoproteins such as FN (reviewed in [76,77], and [39]; [44,61,62,78,79]) which interact forming a mesh-like structure [77,80]. 

The function of the PCM is defined by the presence of type VI collagen, a non-fibrillar collagen that forms a microfibrillar network to anchor the chondrocyte to the ECM [29,30,82]. It integrates with the surrounding ECM by binding to type II collagen, aggrecan and hyaluronan with links to BGN, DCN and type IX collagen [44,78,82,83]. In addition, it binds at numerous sites to the chondrocyte cell membrane via integrin receptors and the chondroitin sulphate proteoglycan neural/glial antigen 2 (NG2) [84,85] and thus mediates cell–matrix interactions facilitating transduction of biomechanical signals from the cartilage ECM (reviewed in [86]). The critical role of type VI collagen has been demonstrated by several studies whereby knockout or knockdown of the *Col6a1* gene results in alterations in PCM structure and function [30,87,88]. Twomey et al. showed that *Col6a1* knockdown results in a reduction in the expression of the genes encoding aggrecan, BGN and DCN during chondrogenesis [88] and knockout of type VI collagen in mice results in a reduction in the PCM elastic modulus [30]. Chondron formation in these mice occurs, but PCM stiffness is reduced resulting in aberrant buffering of loads applied to the cartilage [87]. 

Perlecan also plays an important role in mediating PCM organisation and mechanical stability (reviewed in [89,90]) colocalising with type VI collagen and affecting the PCM modulus [62,91]. It provides a pericellular reserve of inactive fibroblast growth factor 2 (FGF2) [92] which is released following deformation of the PCM and subsequent activation of downstream signalling pathways to modulate ECM homeostasis [73,92,93]. Other growth factors include connective tissue growth factor (CTGF) which is sequestered in a covalent complex with latent TGFβ by perlecan [94] and, more recently, hepatoma-derived growth factor (HDGF) which is heparan sulphate bound in the PCM [75]. The mechanism of FGF and HDGF release is thought to be due to a rapid flux in Na^+^ that is displaced from the highly sulphated aggrecan-rich matrix on tissue compression [75,94]. Perlecan knockdown impacts PCM structure, composition and functional properties [95,96,97]. In mice devoid of perlecan *(Hspg2^C1532Y-Neo^*), encoded by the heparan sulphate proteoglycan-2 (*Hspg2*) gene, both in situ cell and ECM stiffness were significantly reduced which was hypothesised to be attributed to an alteration in ECM deposition resulting from disrupted growth factor signalling, e.g., FGF-2 and FGF-18 [97]. 

BGN and DCN localise to the N-terminal globular domain of type VI collagen [44] controlling its aggregation and are thus critical for the structural integrity of the PCM [74,98]. They act as functional bridges between type II and VI collagen to connect the PCM to the TM/ITM [44] and help control fibrillogenesis and growth factor bioavailability [72,74]. DCN knockdown results in an increase in aggrecan, BGN and sox9 gene expression early in chondrogenesis and prevents the formation of a normal microfibrillar layer surrounding the cell [88]. In DCN null mice (*Dcn*^−/−^), the modulus of the PCM is significantly reduced from 2 weeks of age onwards concomitant with the absence of a maturation-associated stiffening effect [65]; similar effects were also observed in the TM/ITM consistent with the finding that, at the tissue level, DCN loss results in a reduced cartilage modulus at ≥2 weeks of age. Impairment of the PCM because of a loss of DCN also affected Ca^2+^ signalling and thus the chondrocytes response to load [65]. *Dcn*^−/−^ cartilage has a significantly reduced aggrecan component and mild perturbations in collagen fibril nanostructure resulting in inferior biomechanical properties as evidenced by a decreased modulus and elevated hydraulic permeability [99]. Han et al. (2019) hypothesised that DCN is instrumental in increasing the adhesion between adjacent aggrecan molecules, as well as between aggrecan and type II collagen fibrils, and that DCN absence impairs biomechanical function by preventing physical linkages to increase aggrecan adhesion/assembly at the nanoscale level [99]. As well as their structural roles, DCN and BGN also sequester growth factors and signalling molecules. DCN binds TGFβ3, FGF2, tumour necrosis factor alpha (TNFα), platelet-derived growth factor (PDGF) and insulin growth factor 1 (IGF1) acting as a reservoir of biological factors that can subsequently modulate cellular homeostasis [72,74,100]. BGN also acts as a reservoir for the Wnt ligand Wnt3a and participates in canonical Wnt signalling via the cell surface receptor, low-density lipoprotein receptor-related protein 6 (LRP6) [101]. Although release of Wnt3a has not been shown to be triggered by mechanical load, there is evidence that the pathway is activated following injury [102]. Other cartilage ECM molecules including fibrillin and the glycoprotein fibulin localised to the PCM are also reported to sequester growth factors such as TGFβ1 [103]. Sequestration of TGFβ within the matrix is critical for cartilage health [104] since it mediates mechanical responses in cartilage [105] and is a potent inducer of cartilage ECM synthesis and suppressor of catabolism [106,107,108]. Indeed, high levels of TGFβ are found in normal articular cartilage but almost absent in OA [109].

Other ‘basement membrane’ components are also associated with the pericellular matrix including nidogen-1 and -2 [110], laminin α1, α2, α4, α5, b1 and g1 [26,111] and type IV collagen [27,112]. Both nidogen-2 and laminin act as chondrogenic regulators increasing sox9 and promoting type II collagen, COMP and aggrecan synthesis and decreasing type I collagen synthesis [112]. Nidogen-1 and -2 mediate cell-matrix interactions, binding to integrin αV and b1 in vivo [110].

The PCM subsequently integrates with the surrounding tissue, via a basket-like weave of fine collagen fibrils, PGs and FN forming the TM [113]. This chondroitin sulphate-rich matrix protects the chondrocytes against mechanical stresses contributing to the ability of the matrix to withstand substantial loads [39,114]. The TM is located within an interterritorial matrix comprised of fibrillar and non-fibrillar collagens, PGs, laminins and FN which collectively contribute to mechanosensing to regulate chondrocyte function and cartilage homeostasis [115]. Alterations to ECM composition, particularly of PCM properties, can influence cartilage function inducing or progressing tissue degeneration and OA pathology.

## 4. Mechanical Load Influences Articular Cartilage ECM Turnover

Articular cartilage can respond to mechanical perturbations in the local tissue environment, sensing alterations in the magnitude or type of loading applied, and eliciting cellular behaviours to effect change. During normal physiological loading, a homeostatic balance of anabolic and catabolic activities is maintained ensuring the slow but consistent turnover of the PCM. However, following application of abnormal, non-physiological loading (excessive loading, joint trauma or malalignment), the balance of chondrocyte metabolism is disturbed resulting in dysregulation of PCM turnover and enhanced catabolism, cartilage degradation and onset of OA pathology (Figure 2B,C). 

Articular chondrocyte mechanobiology has been investigated extensively over the past few decades, utilising both in vivo and in vitro models to establish the interplay of mechanical loading in conjunction with the structure and function of cartilage. In situ, the chondrocyte resides within a dynamic mechanical environment comprising a combination of compression, hydrostatic pressure, shear stress, osmotic stress and tensile strain. Typically, direct contact between opposing joint surfaces can yield dynamic compressive stresses of 5–6 MPa in the human knee during walking, and dynamic compressive stresses in the knee and hip can reach 10–20 MPa during activities such as climbing stairs [116]. Numerous studies have been undertaken to investigate the response of cartilage chondrocytes to these different loading parameters, whereby defined mechanical forces have been applied to chondrocytes in isolation or in situ and effects on ECM metabolism characterised.

### 4.1. Compression

#### 4.1.1. Dynamic Compression

Maintenance of healthy articular cartilage relies on continuous turnover of the ECM which is achieved through a balance of biosynthetic and degradative events. This is attributed to the application of moderate compressive loads experienced during movement. In vivo, the static and dynamic stresses and strains that articular cartilage is subjected to are dependent on anatomical location within the joint and the specific activity being undertaken [117,118,119,120]. These compressive strains are not uniform through the tissue depth with >50% experienced at the joint surface, 10–20% in the transitional zone and <5% in the deep zone [121]. 

Elucidation of ECM collagen involvement in withstanding dynamic compressive strains (20%, 0.25 Hz) has indicated, at the nanoscale level, that the collagen fibrils exhibit a reversible reduction in the fibrillar D-period promoting a state of ‘pre-strain’ as well as an alteration in the degree of fibril orientation [122]. The reversible reduction in collagen fibril pre-strain has been attributed to a pressure-induced reduction in the hydrostatic swelling pressure in the ECM due to localised loss of water from the ECM. Furthermore, how the chondrocytes respond to dynamic compression is highly dependent on specific parameters including magnitude of load, frequency, strain-rate and loading history. Numerous studies in cartilage explants and chondrocyte-seeded scaffolds have demonstrated that following application of physiological magnitudes of dynamic compression (~10–20%), chondrocytes increase biosynthesis of ECM molecules, including proteoglycans [123,124,125,126], collagens [126,127], FN and COMP [127], which support tissue structure and function. Buschmann et al. also found that tissue regions experiencing high interstitial fluid velocities as a result of dynamic compressive loading correlated with enhanced aggrecan synthesis [125]. Furthermore, intermittent loading regimens were found to be superior in promoting ECM synthesis (reviewed in [128]) contributing to increased proteoglycan synthesis following short loading durations [129] and over several weeks of culture [126]. Chondrocyte–agarose constructs exposed to alternate days of intermittent dynamic stimulation (2.5%) with days of free-swelling culture increased both equilibrium and dynamic moduli demonstrating the importance of physiological loading in promoting superior biomechanical properties of the ECM [130]. One mechanism by which compressive load induces ECM synthesis, in chondrocytes embedded in agarose, is via the regulation of transient receptor potential vanilloid 4 (TRPV4) channel function. TRPV4 is a non-selective Ca^2+^ channel that orchestrates osmotic-pressure-induced ECM turnover [131]. Application of compressive load (10% peak–peak sinusoidal (7% offset), 1 Hz, 3 h/d) over a 4-week period increased ECM accumulation enhancing sGAG and collagen production in chondrocytes, which was negated when TRPV4 activity was inhibited [132]; furthermore, a reduction in the equilibrium Young’s Modulus for the chondrocyte–agarose constructs was observed indicating a role for TRPV4 in promoting mechanically induced ECM biosynthesis and conferring tissue material properties. 

Physiological loading, via voluntary wheel running for 3 weeks, has been shown to regulate the expression of *Prg4* in vivo, in the knee joints of knock-in mice expressing a tamoxifen-inducible Cre recombinase from the *Prg4* locus (*Prg4*^GFPCreERt2/+^; *Rosa26*^floxlacZ/+^ mice) [133]. A doubling in the number of articular chondrocytes expressing *Prg4* was observed in the knee joints following the running regimen, with the greatest increase observed in the SZ cells of the tibial plateau, indicative that mechanical loading can activate downstream intracellular signalling pathways to induce *Pgr4* expression. Ogawa et al. (2014) demonstrated that mechanically induced lubricin expression was mediated in a cyclooxygenase-2 (COX-2)-dependent manner, via increased prostaglandin E2 (PGE_2_) production and phosphorylation of CREB concomitant with nuclear translocation of CREB-regulated transcription coactivator 1 (CRTC1/2) to induce *Prg4* transcription [133].

#### 4.1.2. Static Compression

Depending on the activity and anatomical location within the joint, articular cartilage can also experience static compressive strains in vivo which can influence ECM homeostasis [117,118,119,120]. With sustained compression, the expulsion of water and ions induces indirect physicochemical effects such as streaming potentials, changes in pericellular pH, osmolarity, fixed charge density and osmotic pressure which contribute to inhibiting ECM synthesis. Previous studies have demonstrated a magnitude-dependent reduction in aggrecan [134,135,136], link protein [137] and type II collagen synthesis [134,135,136] with increasing levels and duration of static compression. Intriguingly, hyaluronan synthesis was unaffected by static compression [137] implying that static compression does not inhibit all cellular activity, rather that it contributes to a mechanotransduction mechanism promoting spatial and/or temporal changes in specific ECM molecule biosynthesis. 

Elucidation of ECM collagen involvement in withstanding static compressive strains has indicated at the nanoscale level the existence of a collagen fibril tensile pre-strain of approximately 1–2% [138]. A rapid reduction and recovery of the collagen pre-strain was found to occur during stress–relaxation and approximately 60 s after the onset of peak load (20% static strain); the initial reduction was attributed to disordering of the intrafibrillar molecular packing, concomitant with alterations in the axial overlapping of tropocollagen molecules within the fibril. The presence of this pre-strain phenomena was attributed to the osmotic swelling potential of the PGs as enzymatic removal of the chondroitin sulphate moieties on aggrecan disrupted the collagen fibril pre-strain and its transient response during the stress–relaxation phase [138], demonstrating the equivalent importance of both the collagen fibrils and PG aggregates in enabling cartilage biomechanical functionality. 

### 4.2. Shear Stress

In vivo, cartilage that is regularly subjected to high levels of shear stress, i.e., patellar surface of femur and femoral condyles, exhibits a thicker SZ concomitant with a greater degree of collagen orientation compared to a region that is preferentially subjected to weight bearing, e.g., the tibial plateaus [139]. Fluid shear stress, involving the movement of fluid over a tissue that in vivo can result from pressure of the synovial fluid against the cartilage during joint movement, is known to influence the expression of several key ECM molecules. A systematic review of fluid shear studies demonstrated an overall increased production of type II collagen and aggrecan in chondrocytes under a variety of loading regimens [140]; the reported stimulation of ECM synthesis is likely attributed to improved diffusion and transport of nutrients facilitated by fluid flow. Furthermore, shear stress has been found to influence lubricin expression directly with application of dynamic shear (3%) inducing its production and secretion, the latter effect still evident 3 days following load cessation [141]; shear-induced lubricin production can contribute to the low-friction bearing properties of the cartilage surface during joint movement. Lubricin secretion was also induced following exposure to a steady laminar non-pulsatile shear stress (7.6 dyn/cm^2^) in SZ chondrocytes only [133]; no effect was observed from chondrocytes isolated from cartilage mid-zone substantiating its function in joint surface lubrication. Interestingly, shear-induced lubricin production is mediated via the up-regulation of PGE_2_ and secretion of extracellular ATP via P2X7 and P2Y2 receptor engagement in a Ca^2+^-dependent manner [133].

An alternative form of shear, referred to as tissue or contact shear, is instigated by solid-on-solid contact. Application of tissue shear (3%) was stimulatory inducing both protein and PG biosynthesis in cartilage explants, indicating that chondrocytes can respond to tissue shear stress inducing ECM synthesis in the absence of macroscopic tissue-level fluid flow, via deformation of the chondrocytes and associated PCM [142].

### 4.3. Hydrostatic/Osmotic Pressure

A high GAG content is essential to the load-bearing requirements of joints conferring the tissue with the properties of low hydraulic permeability and high swelling pressure [143]. The high PG content in the cartilage ECM providing negatively charged sGAGs attracts cations to maintain an electroneutral environment and, in so doing, creates an osmotic gradient against the synovial fluid. This osmotic gradient imparts a swelling pressure enabling the PGs to expand within the constraints imposed by the surrounding collagen network. Following joint activity, water is exuded from the compressed cartilage, but during the initial stages of loading, the low hydraulic permeability leads to fluid pressurisation, increasing the hydrostatic pressure within the tissue. Following load removal, water is re-imbibed into the cartilage restoring the tissue’s original physical properties. However, with increasing load magnitude or duration of load application, not only is the tissue subjected to hydrostatic pressure, but the exudation of water and ions can alter the biophysical environment resulting in alterations to local pH and osmolarity, as well as inducing fluid flow and streaming potentials [144]. It remains to be fully elucidated whether in vivo the chondrocytes react to alterations in hydrostatic pressure via tissue deformation or whether they are responding to alterations in the local biophysical environment; evidence from in vitro studies indicates that the chondrocytes are highly sensitive to both direct, i.e., deformation, and indirect signals, i.e., biophysical cues, to effect cartilage turnover. 

For example, ‘physiological’ hydrostatic loading regimens have previously been shown to significantly enhance PG synthesis in cartilage [145] and articular chondrocytes [146,147] and collagen synthesis in chondrocytes [146,147]; however, application of ‘non-physiological’ pressures (i.e., increased magnitude and loading cycle duration) resulted in reduced biosynthetic rates [145,146]. A recent meta-analysis corroborated these findings demonstrating a diversity in chondrocyte response with both anabolic and catabolic behaviours, with a magnitude within the mid-high physiological range of cartilage (5–10 MPa) enhancing PG synthesis [148].

Hydrostatic pressure can also act indirectly by modulating biophysical cues which influence solute concentrations around the resident chondrocytes in the ECM. In vitro studies have shown that chondrocytes respond to changes in osmolarity via transient increases in intracellular Ca^2+^ to effectively regulate ECM synthesis and chondrocyte metabolism [149]. Articular chondrocytes exhibited a frequency dependent intracellular Ca^2+^ transient in response to dynamic osmotic loading culminating in increased aggrecan transcription, mediated via cytoskeletal actin re-organisation [149]. Ca^2+^ inhibitor studies have demonstrated the differential involvement of Ca^2+^ entry pathways in response to static and dynamic hypotonic loading to modulate ECM biosynthesis. Importantly, the TRPV4 channel is not only osmotically sensitive [131] but has also been demonstrated to be activated in response to tensional force, acting on cell-integrin-ECM adhesions in chondrocytes [150]. Further evidence has inferred that mechano-osmotic signalling through TRPV4 activation is mediated through the mechanical properties of the cartilage ECM, specifically type VI collagen in the PCM [31]. In the absence of type VI collagen (*Col6a1*^−/−^ mice), chondrocytes presented a heightened TRPV4-mediated response to hypo-osmotic stress as compared to age-matched wild-type controls at 2 months of age [31]. The observed increase in hypo-osmotic-stress-induced cell swelling was associated with a loss of mechanical properties in the *Col6a1*^−/−^ PCM, suggestive that type VI collagen is important in regulating the mechanical properties of the PCM, cell swelling and osmotically induced signalling mechanisms. Clearly, type VI collagen plays an instructive role in regulating the transduction of mechanical and physicochemical signals from the cartilage ECM to the chondrocyte, via the critical involvement of the PCM [63,151]. 

In addition to TRPV4, TRPV2 has been shown to be important in the maintenance of articular cartilage homeostasis [152]. Cartilage specific knockout of TRPV2 (*Trpv2^−/−^*) in mice resulted in the development of OA and three forms of mechanical stimulation (hypo-osmotic stress, fluid flow, stretch) invoked Ca^2+^ influx via up-regulation of lubricin and activation of CaMKK-Creb signalling which was abolished in *Trpv2^−/−^* chondrocytes [152].

### 4.4. Tensile Strain

In vivo, tensile strains are generated tangential to the articulating joint surface and at the interface between cartilage and bone. During joint articulation, tensile strain is experienced by the articular chondrocytes, principally arising as a consequence of compression-induced cellular deformation, i.e., a reduction in cell height accompanied by an increase in cell width [153]. Chondrocytes subjected to 10% tensile strain in vitro increased *COL1A1* expression concomitant with a significant reduction in *COL2A1* and *ACAN* transcript levels [154]; application of a lesser strain of 5% elicited no effect on the expression of these ECM molecules. This contrasts to our own study where a 7.5% strain increased chondrocyte type II collagen and aggrecan mRNA levels 4 h following strain cessation [155]. Not surprisingly, tensile strains are found to elicit varying chondrocyte responses which is largely dependent on the magnitude and frequency of strain applied (comprehensively reviewed in [156]).

A systematic review of the literature investigating the influence of cyclic tensile strain (CTS) on chondrocyte metabolism identified that, overall, longer loading durations (3–6 h) increased type II collagen and aggrecan transcript levels [156]; however, this was strain-magnitude-dependent with enhanced transcription of ECM molecules at higher strains (10–24%). Extended loading episodes (16–72 h) were also found to be detrimental to matrix synthesis, resulting in transcriptional inhibition of type II collagen and aggrecan. Such biosynthetic patterns were also largely evidenced at the protein level (reviewed in [156]). Interestingly, the biosynthetic response was dependent on the ECM substrate the chondrocytes were cultured on; chondrocytes seeded on FN enhanced PG synthesis in response to 7% tensile strain [157]; seeding on type I collagen elicited no response indicating how important integrin-mediated attachment of chondrocytes to their native ECM is in transmitting mechanical signals.

Other ECM molecules are also regulated by tensile strain, with 5–9% strain increasing transcript levels of lubricin (<48 h [158]), FN (<24 h [159]) and matrilin-1 (<48 h [160]). A concomitant induction of lubricin protein was also observed followed a 7% strain stimulus, but a significantly higher strain (21%) inhibited its expression [161], illustrating the concept that moderate loading facilitates joint lubrication and low friction movement, whereas excessive loading impacts lubricin production and functionality. Furthermore, lubricin and COMP mRNA levels were also elevated in response to 9% tensile strain in chondrocytes embedded in 3D alginate constructs [161], illustrating the critical role of the mechanical stimulus in effecting changes to ECM composition to promote joint function. 

In matrilin-1-deficient (*Matn1*^−/−^) chondrocytes, strain-induced transcription of *Acan* and *Col2a1* was abolished [162]. Furthermore, load-induced downstream activation of Indian Hedgehog (Ihh) signalling was suppressed in engineered chondrocytes devoid of functional matrilin-3 [160], indicating the importance of this PCM molecule in mediating mechanotransduction. Interestingly, overexpression of the matrilins also adversely affected chondrocyte mechano-signalling, which was attributed to a likely direct impact on PCM mechanical properties or a possible saturation of ECM binding sites by their ligands [160]. Such evidence suggests that load-mediated modification of PCM composition has the potential to modify the ‘threshold’ of chondrocyte mechanosensing, i.e., functional adaptation to load applied. 

Under tensile load, cartilage is reportedly much stiffer in the SZ compared to the deep zone [163]. Using the technique of polarisation sensitive second harmonic generation (P-SHG), the relationship between macroscopic strain applied to cartilage and the sub-micron response of the collagen fibrils was investigated [164]. As applied strain increases (0–16%), orientation of the collagen fibrils preferentially aligned with the direction of strain and became more organised in the SZ of cartilage; this increase in local organization of the collagen fibrils in the SZ weakly correlated with tensile modulus suggestive that higher forces are required to generate these changes in situ [164].

### 4.5. Injurious Loads

**Figure 4:**
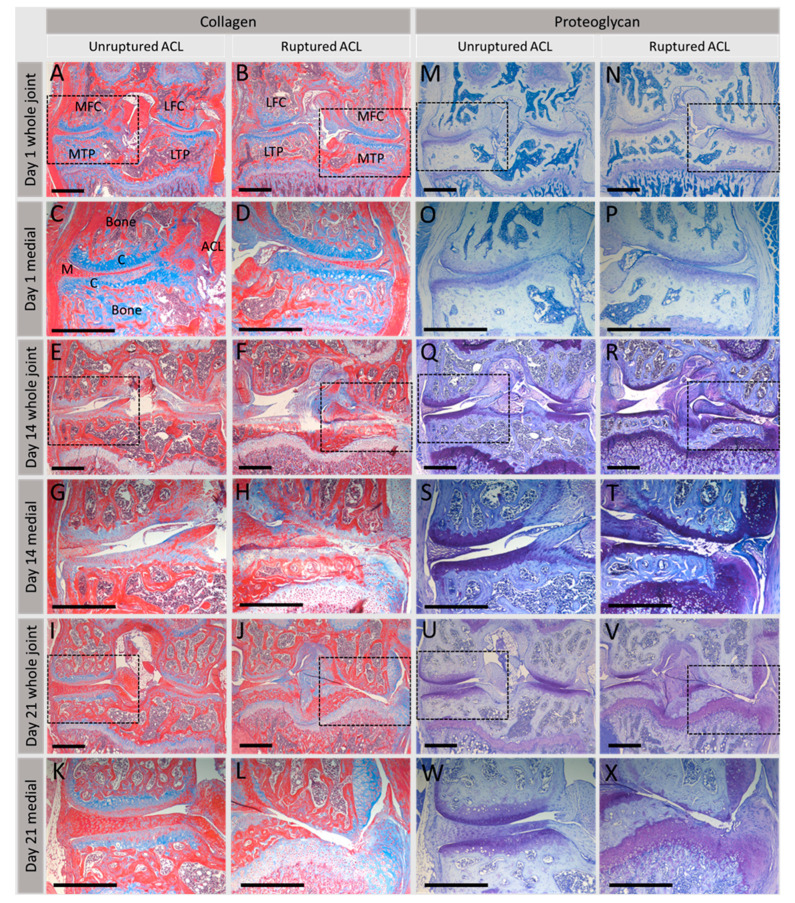
Effect of injurious load on articular cartilage integrity in the synovial joint. Collagen and proteoglycan distribution in an in vivo non-surgical murine model of post-traumatic OA following rupture of the anterior cruciate ligament (ACL) [167]; knee joint integrity was assessed at days 1, 14 and 21 post-rupture and compared to mice with an intact ACL, i.e., unruptured controls. (**A**–**L**) show collagen (blue) distributed throughout cartilage and bone. On day 1, collagen is located evenly throughout cartilage (**A**–**D**); however, at day 14 post-rupture (F and H), cartilage fibrillation is evident with loss of collagen, followed by complete loss by day 21 (**J**,**L**). Proteoglycan distribution (dark blue, (**M**–**X**)) shows a similar distribution throughout the cartilage, evenly distributed at day 1 (**M**–**P**) but gradual loss as joint damage begins to occur at 14 (**R**,**T**) and 21 days (**V**,**X**) post-ACL-rupture. Trichrome stain kit (Abcam, ab150686) was used for collagen staining, toluidine blue for proteoglycans. MFC, medial femoral condyle; LFC, lateral femoral condyle; MTP, medial tibial plateau; LTP, lateral tibial plateau; M, meniscus; C, cartilage. Dashed boxes indicate higher magnification medial compartment images directly below. Scale bars: 500 μm.

Upon injurious loading induced by non-physiological loading or an acute traumatic injury, a key feature is the early inhibition of PG synthesis concomitant with the rapid loss of endogenous PGs from the ECM proceeded by inhibition of collagen synthesis and degradation of the collagen network (Figure 4). In vitro studies have demonstrated that abnormal, i.e., super-physiologic loading magnitudes (>20% compressive strain) do not induce ECM synthesis [127], whilst static or very low frequency loading inhibits ECM synthesis [134]. However, injurious compression shifts cartilage chondrocyte-mediated ECM metabolism to a catabolic phenotype with a magnitude-dependent increase in tissue swelling, GAG loss, degradation of the collagen fibril network and a decreased explant dynamic stiffness [165,166]. 

Not only does injurious load impact PG and collagen production, it also regulates the synthesis and/or secretion of other molecules necessary for articular cartilage function. Secretion of lubricin, essential in facilitating boundary lubrication to provide a low coefficient of friction, was found to be increased from the uppermost region closest to the articular surface following mechanical injury (50% compressive strain applied at a strain rate of 100%/s) which was observed at the gene level too [168]; increased lubricin expression was found to be a transient response to injurious compression [168]. Concomitant with increased lubricin secretion, injurious load induced cell death, altered tissue architecture and reduced PG and collagen content. Furthermore, the cartilage explants displayed an increased coefficient of friction demonstrating that injurious load significantly compromises tissue function [168]. Application of 60 cycles of injurious compressive load (20 and 50 N) on rat knees in vivo resulted in a transient reduction in lubricin within the lesions that formed on the lateral femoral condyles [169]. The detrimental effects of high-magnitude, high-strain-rate injurious loading regimens are thought to result as a consequence of cellular damage, such as chondrocyte apoptosis and necrosis [170]. Interestingly, following injurious load, the surviving cell population lacks a biosynthetic response to physiologic levels of dynamic loading [166], indicative of a sustained modification of chondrocyte mechanotransduction. 

In vivo models have also provided considerable evidence for the consequence of injurious load on cartilage ECM metabolism. Significant, early loss of PGs was observed in our in vivo non-surgical murine model of joint degeneration whereby a single compressive load ruptures the anterior cruciate ligament rupture (ACL) inducing abnormal loading of the joint cartilage (Figure 4) [167]. Following mechanical injury, localised inflammation preceded the extensive loss of ECM proteoglycans and collagen that was observed over time, replicating a post-traumatic osteoarthritis (PTOA) phenotype. 

This dramatic reduction in cartilage ECM content following mechanically mediated joint injury has been correlated with significant effects in the PCM. Using the destabilisation of the medial meniscus (DMM) murine PTOA model, Chery et al. (2020) observed a decrease in the mechanical properties of the PCM, although PCM composition was maintained; type VI collagen, perlecan and BGN remained localised to the PCM at 8 weeks, concomitant with reduced aggrecan content [171]. A significant reduction in the indentation modulus was observed as early as 3 days post injurious load suggestive that PCM degradation began quickly after this insult and preceded changes in the further-removed territorial and interterritorial ECM. However, over the 8 weeks period, the progressive reduction in indentation modulus for the PCM was also detected in the other regions of the ECM. Thus, alterations to the PCM induce a positive feedback loop that reduces protection to chondrocytes and induces OA [171].

An accelerated OA phenotype was reported in both DCN-null (*Dcn^−/−^*) and inducible DCN knockout (*Dcn^iKO^*) mice subjected to medial meniscus destabilisation [172]. Induction of DCN ablation following skeletal maturity to prevent confounding influences of joint growth on outcome measurements demonstrated that DCN absence (*Dcn^iKO^*) accelerated sGAG loss accompanied by the formation of surface fissures but did not significantly affect indentation modulus [172]. DCN loss accelerated aggrecan depletion, thereby impairing aggrecans’ protection of the collagen fibrillar network following mechanical insult; these findings indicate the importance of DCN in promoting ECM structural integrity by providing a ‘physical linker’ to increase molecular adhesion between the ECM components. 

Interestingly, following PTOA induction (DMM model) in inducible BGN (*Bgn^iKO^*) mice, the degree of OA was comparable to control mice and not as severe as the phenotype observed in the (*Dcn^iKO^*) mice [173], implying that BGN loss has a lesser impact than DCN on DMM-induced cartilage degeneration. Utilisation of the DCN/BGN compound (*Dcn/Bgn^iKO^*) knockout mouse in this study indicated that following DCN loss, the accompanying loss of BGN did not aggravate OA progression. However, a significantly higher modulus was reported in the *Dcn/Bgn^iKO^* mice after meniscal destabilisation which was attributed to greater cartilage erosion [173]. The differential phenotypes following DCN or BGN ablation may be explained by differences in their localisation, binding activities and response to joint destabilisation and inflammation; furthermore, although BGN is critical in maintaining cartilage PCM integrity, its concentration and distribution is unaffected in the DMM model [171] suggesting that it does not contribute significantly to OA pathogenesis following meniscal destabilisation. 

The initiating injurious mechanical stimulus clearly influences the behaviour of the tissue and the resultant downstream biosynthetic responses within the cartilage; interestingly, *Dcn^−/−^* mice were protected from the development of OA induced by forced exercise through treadmill running [174]. Reduced PG staining was only observed in the SZ, and the characteristic surface roughening and fibrillation observed in the wild-type mice was absent in the *Dcn^−/−^* mice. This evidence would initially suggest that DCN deficiency improves biomechanical functionality, protecting against cartilage degeneration; however, increased protein levels of TGFβ1, *Chst11* (sulphotransferase enzyme which catalyses formation of *N*-acetylgalactosamine-4-sulphate in chondroitin sulphate moieties) and chondroitin-4-sulphate were also observed in *Dcn^−/−^* mice following forced exercise [174]. The TGFβ superfamily members, which DCN can bind and functionally modulate, are known to be imperative in regulating cartilage homeostasis [175]. Therefore, it has been hypothesised that a compromised sequestration of TGFβ1 in the *Dcn^−/−^* mice enhances active TGFβ1 availability, promoting sulphation of GAGs and imbibition of water, to augment tissue biomechanical properties and delay load-induced cartilage degeneration [174]. The divergence in response of *Dcn^−/−^* mice to abnormal loading such as forced running versus meniscal destabilisation might be due to the induction of an initial inflammatory response in the DMM model, which is absent following forced exercise; however, this remains to be confirmed. 

Utilising an alternative model of PTOA, involving the transection of the anterior cruciate ligament and medial collateral ligament, in tenascin-C (*Tnc*^−/−^) knockout mice, the extent of cartilage degeneration was accelerated relative to wild-type mice, suggestive that tenascin-C is ordinarily involved in protecting against cartilage destruction [176].

Induction of DMM in matrilin-1-deficient mice (*Matn1^−/−^*) significantly reduced *Acan* and *Col2a1* transcript levels, concomitant with greater erosion of the femoral cartilage relative to DMM-induced wild-type mice [162]; the pattern of erosion differed between the mice with irregular and substantial degenerative ECM changes predominating in the *Matn1^−/−^* mice, along with more significant ECM disorganisation. Interestingly, these histological effects were not observed in uninjured *Matn1^−/−^* mice indicating that the mechanical insult initiates cartilage degeneration and that the *Matn1^−/−^* chondrocytes are incapable of adapting to this injurious load [162].

Injury and deleterious load results in the release of sequestered growth factors from the PCM followed by activation of downstream signalling pathways that modulate ECM homeostasis [93]. FGF2, HDGF and CTGF are all released upon injurious cutting of cartilage [75,92,94]. In addition, supra-physiological compression [177] and tissue shear [105] also promote latent TGFβ1 release from cartilage rapidly inducing Smad signalling which can impact ECM biosynthesis [175]. A recent study has shown that compression-induced growth factor release occurs preferentially in areas of low matrix stiffness such as from the PCM in the SZ resulting in SMAD2 phosphorylation indicative of TGFβ activation [75]. The study found that growth factor release following injury was due to a shift in the matrix Na^+^ concentration and hypothesised that the SZ selectively loses water, causing a local increase in the concentration of free Na^+^, and this drives growth factor release when compression is applied. These findings would explain why OA cartilage loses its ability to repair due to the severe loss of PG and the subsequent inability to release growth factors upon injury. Interestingly, the absence of loading rapidly reduced TGFβ1 signalling in chondrocytes, thought to be due to effective sequestration of TGFβ1 in the ECM, which could be rescued upon the re-introduction of dynamic compression [178], intimating how ECM components can self-regulate cartilage ECM homeostasis. 

Irrespective of the loading stimulus applied to articular cartilage, the mechano-signalling process is reliant on stimulation of mechanoreceptors localised to the chondrocyte membrane including integrin receptors, ion channels and primary cilia (reviewed in [179]). More recently, mechanically activated Piezo ion channels, Piezo1 and 2, have accrued much interest following reports of their involvement in chondrocyte mechanotransduction in response to hyper-physiological and injurious loads [180,181,182]. Following mechanically induced ECM deformation and stimulation of cell surface mechanoreceptors, activation and/or suppression of intracellular signalling pathways elicit downstream events to regulate chondrocyte response; this is beyond the scope of the current review but has been extensively discussed in [179,183,184]. 

## 5. Bidirectional Reciprocity Involves Alterations to the ECM Due to Mechanically Mediated Cell Behaviours

The PCM is instrumental in transducing biomechanical signals to the chondrocytes, influencing cell behaviour such as metabolism, inflammatory cascades and intracellular signalling events to effect ECM remodelling. Irrespective of the loading stimulus applied to articular cartilage, the mechano-signalling process is reliant on stimulation of mechanoreceptors localised to the chondrocyte membrane including integrin receptors, ion channels and primary cilia (reviewed in [179]). More recently, mechanically activated Piezo ion channels, Piezo1 and 2, have accrued much interest following reports of their involvement in chondrocyte mechanotransduction in response to hyper-physiological and injurious loads [180,181,182]. Following receptor engagement and/or activation of ion channels, the biomechanical signal is transduced via assembly of focal adhesion complexes involving talin, vinculin and other structural and scaffolding proteins which result in cytoskeletal network re-organisation [185,186]. It has been well documented that the cytoskeleton acts as a conduit connecting the ECM to the nucleus, thereby facilitating dynamic and reciprocal interactions between the matrix and the nucleus to orchestrate gene expression and regulation of tissue homeostasis [187]. Re-organisation of the actin cytoskeleton has been shown to activate a number of downstream signalling cascades including Rho GTPases [188,189], mitogen-activated protein kinases (MAPKs) [190,191,192], phospholipase C [193] and calcium/calcineurin [149,194,195] to name but a few which converge in the nucleus to induce transcriptional responses that influence cell behaviours, PCM/ECM remodelling and ultimately tissue properties. Active Rho GTPase signalling results in the nuclear translocation of mechanosensitive Yes-associated protein (YAP) and transcriptional co-activator with PDZ-binding motif (TAZ) to initiate transcriptional events in the regulation of tissue homeostasis [189,196]. 

Furthermore, studies have implicated a role for the nucleus in directly transducing a biomechanical signal via the linker of nucleoskeleton and cytoskeleton (LINC) complex [197]. The LINC complex, localised to the nuclear membrane, binds to all three cytoskeletal proteins and in particular to the intermediate filament lamin A which anchors chromatin to the nuclear lamina (comprising lamins A and C) [198] providing a structural pathway for force transmission from the cell surface to the chromatin [199]. As lamins A and C are located at the nuclear periphery, in addition to the nuclear interior, they associate with both hetero- and transcriptionally active euchromatin [200] providing a mechanism by which mechanical load can regulate gene transcription [201]. Increased expression of type II collagen [126,154,156,161], aggrecan [123,124,125,154,156], other PCM/ECM molecules [133,158,159,160,161] and anabolic growth factors, e.g., TGFβ1 [75,92,94,175], has been demonstrated in response to physiological load, whereas non-physiological load induced expression of pro-inflammatory cytokines [167,202,203] and proteolytic enzymes [167,202,204]. Clearly, mechanically mediated transcriptional induction of key mediators of either chondrocyte matrix biosynthesis or degradation influences the extent of cartilage remodelling which has the potential to impact tissue properties and thus the bidirectional reciprocity of the chondrocytes with their surrounding PCM/ECM environment. Activation and/or suppression of specific intracellular signalling pathways that elicit downstream events to regulate chondrocyte response is beyond the scope of the current review but has been extensively discussed in [179,183,184].

## 6. Consequences of Articular Cartilage ECM Degeneration on Mechanical Integrity

Interaction of ECM molecules with chondrocytes regulates cell behaviour and the remodelling process; furthermore, proteolytic degradation of cartilage ECM components generating breakdown fragments can, in turn, activate or inhibit additional signalling pathways. Reciprocal engagement of the cells with the surrounding ECM can thus impact tissue homeostasis and, if this process becomes dysregulated, results in cartilage pathology. Mechanical loading has been reported to increase expression of proteolytic enzymes, including the matrix metalloproteinases (MMP) and aggrecanases (ADAMTS) responsible for degradation of collagen and PGs in the ECM [205]. Expression and activation of the collagenases MMP 1 and 13 were increased in vivo in cartilage exposed to supra-physiological compression [206] and excessive running [207], correlating with in vitro observations demonstrating enhanced expression in chondrocytes exposed to increased fluid shear [208]. 

### 6.1. Mechanically Mediated Degradation of Key ECM Components Can Result in Accumulation of Bioactive Breakdown Fragments 

One of the consequences of enhanced proteolysis is the generation of bioactive breakdown fragments. Key ECM breakdown fragments detected in the cartilage matrix become endogenous catabolic factors activating pro-inflammatory pathways and potentiating further joint damage [209] (reviewed comprehensively in [210]). These extracellular damage-associated molecular pattern molecules (DAMPs) include those derived from type II collagen [211], fibromodulin [212], hyaluronan [213], tenascin C [214], aggrecan [215] and FN [204,216]. DAMPs are increased in knee OA compared to the hip and are therefore thought to be generated due to increased mechanical stress on the joint [217]. They are also released following impact loading of chondrocytes [170]. The biological activity of these DAMPs goes through pattern recognition receptors including Toll-like receptors (TLRs) to initiate downstream signalling cascades leading to the activation of several transcription factors, such as NFκB, key regulators of the inflammatory response. The downstream consequence of this is the release of matrix-degrading enzymes such as MMPs and ADAMTSs and pro-inflammatory cytokines. Degradative fragments of type II collagen occur in normal and in OA cartilage and modulate the expression of several MMPs in articular cartilage chondrocytes with the potential to initiate catabolic pathways [211]. Injurious loading, via a single impact trauma to cartilage in vitro, induces the production of FN fragments concomitant with increased MMP3 levels, potentiating a positive feedback loop to sustain cartilage ECM damage [204]. Increased FN fragment levels have also been detected in the cartilage and synovium of patients with OA [218,219]. Catabolism of SLRPs has also been linked to compromised articular cartilage integrity with an increase in the release of soluble, fragmented BGN into the synovial fluid in late-stage OA being reported [220]. This was shown to accelerate PG and collagen loss in cartilage in vitro through elevated NFκB activities, an effect not observed with DCN. Melrose et al. also reported increased fragmentation of SLRPs including DCN, BGN, fibromodulin and lumican in OA knee tissue [221]. Interestingly, a recent study has shown that an aggrecan 32-mer fragment directly activates TLR2 on joint nociceptors and is an important mediator of the development of OA-associated joint pain [222]. Lee et al. also found that an aggrecan 32-mer fragment increased *Mmp13* and *Adamts5* mRNA expression and decreased *Col2A1* and *Acan* mRNA through TLR-2- and NFκB-dependent signalling [215]. Clearly, mechanically mediated degradation of key ECM components can result in accumulation of bioactive breakdown fragments and has the potential to initiate pro-inflammatory signalling pathways, further exacerbating cartilage degeneration and loss of functionality. 

### 6.2. Mechanically Mediated Inflammation Promotes Cartilage Degeneration

Both mechanical injury and the generation of bioactive breakdown fragments can induce an inflammatory response in the cartilage resulting in tissue degeneration. Furthermore, the invoking of a local inflammatory response in combination with continued application of injurious loading can exacerbate this degradative phenotype. Early in vitro studies demonstrated that PG loss induced by injurious mechanical compression was markedly increased when cartilage was simultaneously exposed to pro-inflammatory cytokines, i.e., interleukin-1α (IL-1α) or TNFα, indicative of a synergistic interaction in inducing cartilage degradation [223]. Other studies have also reported on the synergistic interaction of mechanical injury with TNFα and IL-6 with its soluble receptor (sIL-6R) in mediating PG degradation in cartilage [203]; the combination of mechanical injury and TNFα only induced IL-6 production in chondrocytes, the expression of which was found to contribute significantly to the PG loss observed, and clearly evidenced how such a response can propagate ‘follow-on signalling loops’ initiated through interplay of mechanical and cytokine stimulation [203]. Induction of IL-6 following mechanical injury was also observed in cartilage from two separate in vivo models of PTOA studies, concomitant with elevated levels of both aggrecanase and MMP transcripts [167,202]. 

Interplay of mechanical injury and cytokines is pivotal to cartilage degeneration. Inhibition of IL-1 signalling, via administration of an IL-1 receptor antagonist (IL-1Ra), abrogated the inhibitory effect of injurious compression (static) on PG synthesis in cartilage in vitro [224]. Intra-articular administration of IL-1Ra in a murine model of PTOA (moderate closed articular fracture of tibial plateau) significantly reduced serum IL-6 concentrations and inhibited cartilage degeneration and synovial inflammation in vivo [225], substantiating a role for mechanical-injury-mediated inflammation in promoting cartilage matrix catabolism.

Recently, a putative mechanism of action for IL-1 engagement has been postulated to involve TRPV4 activity; dynamic compression induced transcription of IL-1R was suppressed following exposure to a TRPV4 agonist (and enhanced following TRPV4 antagonist) in ATDC5 chondrocytes [226]. It has been hypothesised that excessive compressive stress may impair TRPV4 function via regulation of cell volume, resulting in increased production of IL-1R, as well as ADAMTS-4 transcription, potentially initiating PG loss and adversely affecting interstitial osmotic pressure [226]. Furthermore, as TRPV4 has been shown to be intimately involved with type VI collagen in the PCM [31], PCM mechanical properties might subsequently be affected.

Interestingly, moderate dynamic compression was found to counteract the degradative behaviours observed in vitro when cartilage was exposed to injurious load in combination with the cytokines TNFα and IL-6 with its soluble receptor (sIL-6R) [227]; dynamic strains <20% inhibited the pro-catabolic response of cartilage to injurious load and cytokine challenge by abolishing sGAG loss and aggrecanase activity [227]. More recently, similar observations were reported for the in vitro application of dynamic tibial axial loading (TAL) to counteract the inflammatory response to injurious impact with significant suppression of IL-1β and TNFα transcription mediated by injurious impact [228]. Such studies demonstrate the potential of using physiological loading regimens to mitigate the degenerative phenotypes induced by injurious mechanical loads, thereby conferring protection to cartilage structure and functionality. 

### 6.3. Effect of ECM Degeneration on Mechanical Properties of Cartilage

**Figure 5:**
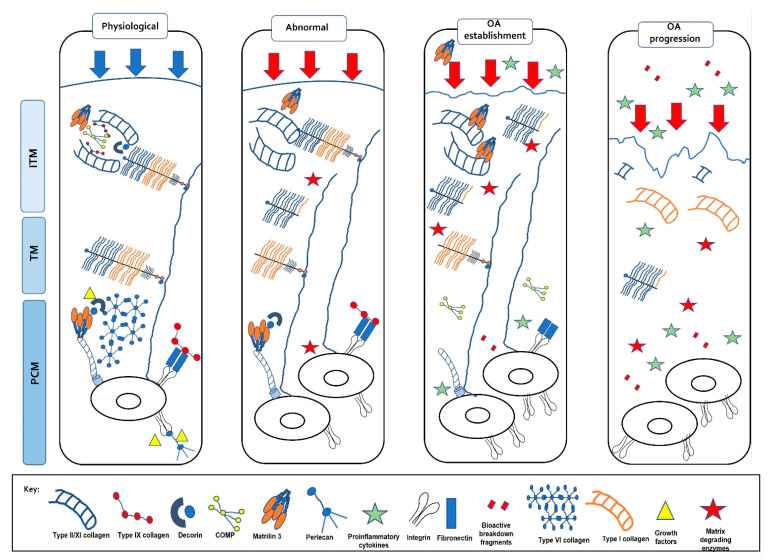
Changes in the extracellular matrix with load. Normal, physiological loads maintain the ECM homeostasis providing the correct biochemical composition which confers its functional properties. Abnormal, pathophysiological loads result in changes in the matrix such as proteoglycan degradation, a loss of the NC4 domain on type IX collagen, reduced type VI collagen and increased type II collagen fibril diameter. This change in matrix compromises the functional properties of the cartilage resulting in OA establishment. Matrilin-3 and COMP increase, pro-inflammatory cytokines and matrix-degrading enzymes are up-regulated, and a switch to type I collagen is observed resulting in OA progression.

An intact ECM has been shown, by several studies, to be critical for conferring cartilage mechanical properties with degenerated cartilage resulting in decreased tensile modulus [229] and decreased shear modulus [230]. Application of compressive load to in vitro cartilage explants enzymatically depleted of either collagen or PGs had little effect on strain distribution, as measured using displacement-encoded MRI during cartilage-on-cartilage cyclic compression [231]. However, depletion of both molecules significantly increased axial, transverse and shear strain components, particularly in the SZ of the medial compartment contact region [231]. Loss of PGs, fibrillation of the collagen network and a switch from type II to type I collagen production in OA (reviewed in [232]) compromises the compressive and tensile properties of the tissue [233]. Interestingly, alterations in the biomechanical properties of knee joint cartilage from *Col9a1^−/−^* mice have been reported; decreased cartilage moduli in knee joint cartilage under both compression (E) and swelling configurations reflected a loss of matrix stiffness by 6 months of age in *Col9a1^−/−^* mice. Loss of matrix stiffness suggests damage to the collagen fibrillar network that may compromise the ability of cartilage to sustain the high stresses and strains associated with joint loading [234]. These findings are similar to those reported for small animal models of OA including a decreased indentation stiffness of humeral head cartilage in *Col2a1^+/–^* mice [235] and modest decreases in the uniaxial modulus of tibial plateau cartilage in *Col11a1^cho/+^* mice [236]. OA chondrons have low pericellular type IX collagen [39], and a loss of type IX collagen with age [29] has been reported which is likely to contribute to the compromised biomechanical integrity of cartilage tissue in OA. This demonstrates the importance of interplay between these ECM molecules in withstanding load and how compromising tissue composition can adversely affect mechanical characteristics perpetuating an OA phenotype (Figure 5). 

With OA, structural and biochemical profiles of the PCM are altered, and degeneration of the PCM is reportedly one of the first events to occur resulting in aberrant chondrocyte mechanotransduction and ultimately cartilage degradation [171,237,238,239]. Using different micromechanical testing methods such as micropipette aspiration or atomic force microscopy (AFM) indentation, studies have revealed that the elastic modulus of the PCM is reduced by 30–50% in OA cartilage [237,240,241]. A net increase in the biosynthesis and deposition of type VI collagen with altered microfibrils [242] results in abnormal swelling and an increase in chondron volume [243]. AFM-based elastic mapping revealed significant alterations in both PCM (30% decrease) and ECM (45% decrease) elastic moduli in the medial condyle of OA joints concomitant with an enlarged biomechanical footprint of the PCM [237]. PCM tissue stiffness reduced with changes in spatial patterns of chondrocytes and a reduction in type VI collagen and perlecan correlating with progression of cartilage degeneration in disease [239]. PCM regions rich in perlecan exhibit lower elastic moduli than regions lacking perlecan, and enzymatic removal of HS chains from perlecan with heparinase III increases the PCM elastic moduli both overall and locally in interior regions rich in both perlecan and type VI collagen [91]. Systemic inhibition of ECM remodelling, using a broad-spectrum MMP inhibitor (GM6001), which also reduces aggrecanase activity in the DMM model, preserved the micromodulus of the PCM [171]. Specifically, inhibition of the early injurious load-induced proteolytic degradation of the PCM prevented detrimental alterations to the micromechanical properties of the cartilage matrix, thereby retaining PCM integrity, protecting normal chondrocyte mechanosensing and supporting tissue homeostasis/joint function. Studies in *Dcn^−/−^* mice substantiate these findings demonstrating that impairment of PCM properties, due to DCN loss, disrupted chondrocyte mechanotransduction as measured by reduced [Ca^2+^]_i_ signalling in response to osmotic stress, which was more pronounced with age (>2 weeks old) [171]. Interestingly, enzymatic removal of the chondroitin sulphate moieties from aggrecan reduced [Ca^2+^]_i_ signalling in wild-type mice which was further compounded in the *Dcn^−/−^* mice [171]; this demonstrated that the contribution of aggrecan to maintaining a fixed charge in the matrix contributes to the ability of the chondrocyte to sense mechanical loads and elicit early [Ca^2+^]_i_ signalling responses. Within the ECM, aggrecan and its chondroitin sulphate sGAG moieties provide the net negative charge facilitating sequestration of cations such as Ca^2+^ for the subsequent activation of ion channels and transmission of mechanical signals. Without these biophysical properties, cartilage chondrocyte mechanotransduction would be severely compromised.

## 7. Concluding Remarks

Articular cartilage responds to mechanical perturbations in the local tissue environment, sensing alterations in the magnitude or type of loading applied, and eliciting cellular behaviours to effect change. In situ, the chondrocytes reside within a dynamic mechanical environment comprising a combination of compression, hydrostatic pressure, shear stress, osmotic stress and tensile strain. Numerous studies have demonstrated the pivotal nature of cell–matrix interactions and the distinct properties conferred by the PCM in regulating the chondrocytes’ mechanical environment, influencing cartilage homeostasis and joint health. Specific PCM components, e.g., type VI collagen, perlecan and SLRPs, are instrumental in facilitating the sensing of mechanical signals and orchestrating appropriate mechano-signalling responses. Insight into composition and structure of the PCM, in conjunction with its mechanical properties in cartilage health and pathology, has been instrumental in our understanding of the reciprocity of the PCM/ECM and chondrocyte mechanotransduction. Furthermore, such studies have revealed the complex interplay of loading, inflammation and growth factors on the biochemical and biomechanical hierarchy of articular cartilage. This knowledge can be effectively utilised to guide development of small molecule therapeutics to prevent early remodelling of the PCM, induced by injurious loading, to delay or halt PTOA progression. Furthermore, understanding how physiological loading promotes PCM/ECM synthesis resulting in deposition of a highly organised matrix will aid development of cell constructs containing the hierarchical structure of cartilage for use in tissue-engineering strategies. Although we do not have a complete understanding of chondrocyte behaviour in response to physiological and non-physiological loading, this review demonstrates a clear bidirectional reciprocity between the chondrocyte and the PCM/ECM in regulating structural, biomechanical and functional characteristics of articular cartilage and how this can impact tissue health and pathology.

## Abbreviations

ACL	anterior cruciate ligament
ADAMTS	a disintegrin and metalloproteinase with thrombospondin motifs
AFM	atomic force microscopy
BGN	biglycan
COMP	cartilage oligomeric protein
COX-2	cyclooxygenase-2
CRTC1/2	CREB-regulated transcription coactivator 1
CTGF	connective tissue growth factor
CTS	cyclic tensile strain
DAMPs	damage-associated molecular pattern
DCN	decorin
DMM	destabilisation of the medial meniscus
ECM	extracellular matrix
FGF	fibroblast growth factor
FN	fibronectin
GAG	glycosaminoglycan
HDGF	hepatoma-derived growth factor
HSPG2	heparan sulphate proteoglycan 2
IGF1	insulin growth factor 1
Ihh	Indian Hedgehog
IL	interleukin
LINC	linker of nucleoskeleton and cytoskeleton
LRP6	lipoprotein receptor-related protein 6
ITM	interterritorial matrix
MAPK	mitogen-activated protein kinase
MMP	matrix metalloproteinase
NG2	neural/glial antigen 2
OA	osteoarthritis
PCM	pericellular matrix
PDGF	platelet-derived growth factor
PG	proteoglycan
PGE_2_	prostaglandin E_2_
PRG4	proteoglycan 4
P-SHG	polarisation sensitive second harmonic generation
PTOA	post-traumatic osteoarthritis
sGAG	sulphated glycosaminoglycan
SLRP	small leucine-rich proteoglycan
SZ	superficial zone
TAL	tibial axial loading
TAZ	transcriptional co-activator with PDZ-binding motif
TGF	transforming growth factor
TLRs	Toll-like receptors
TM	territorial matrix
TNFα	tumour necrosis factor alpha
TRPV	transient receptor potential vanilloid
YAP	Yes-associated protein
